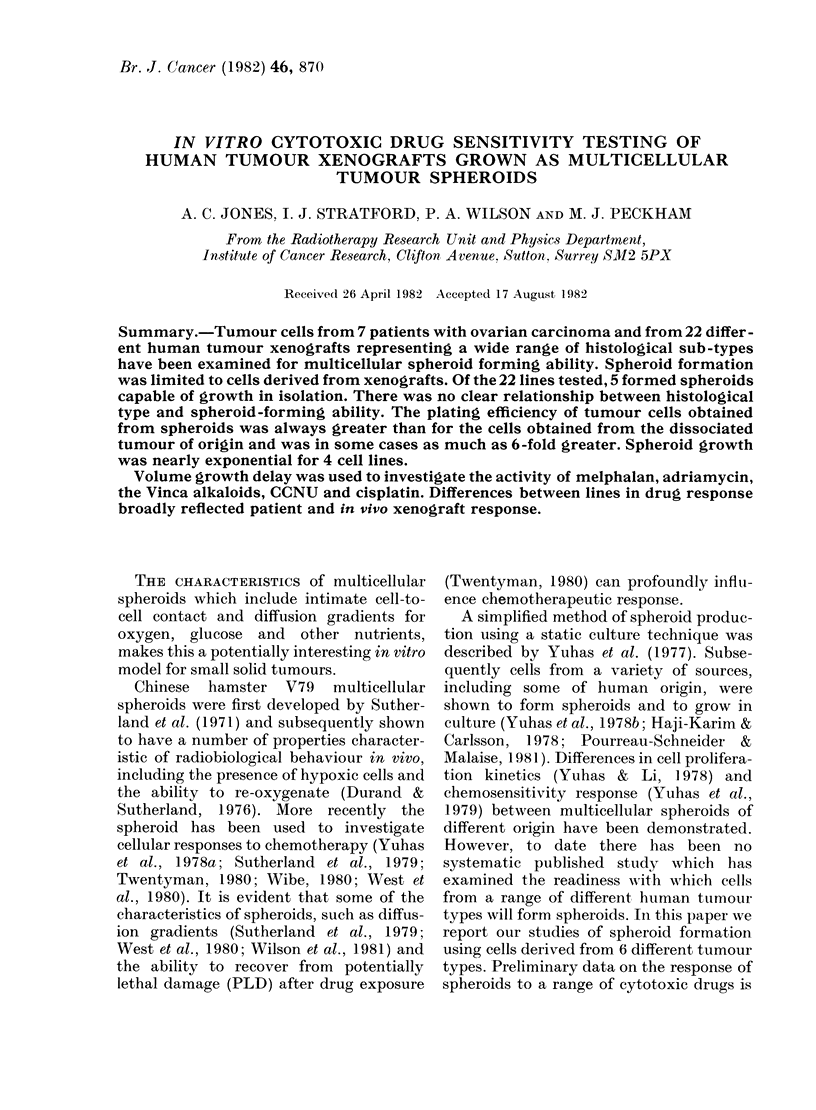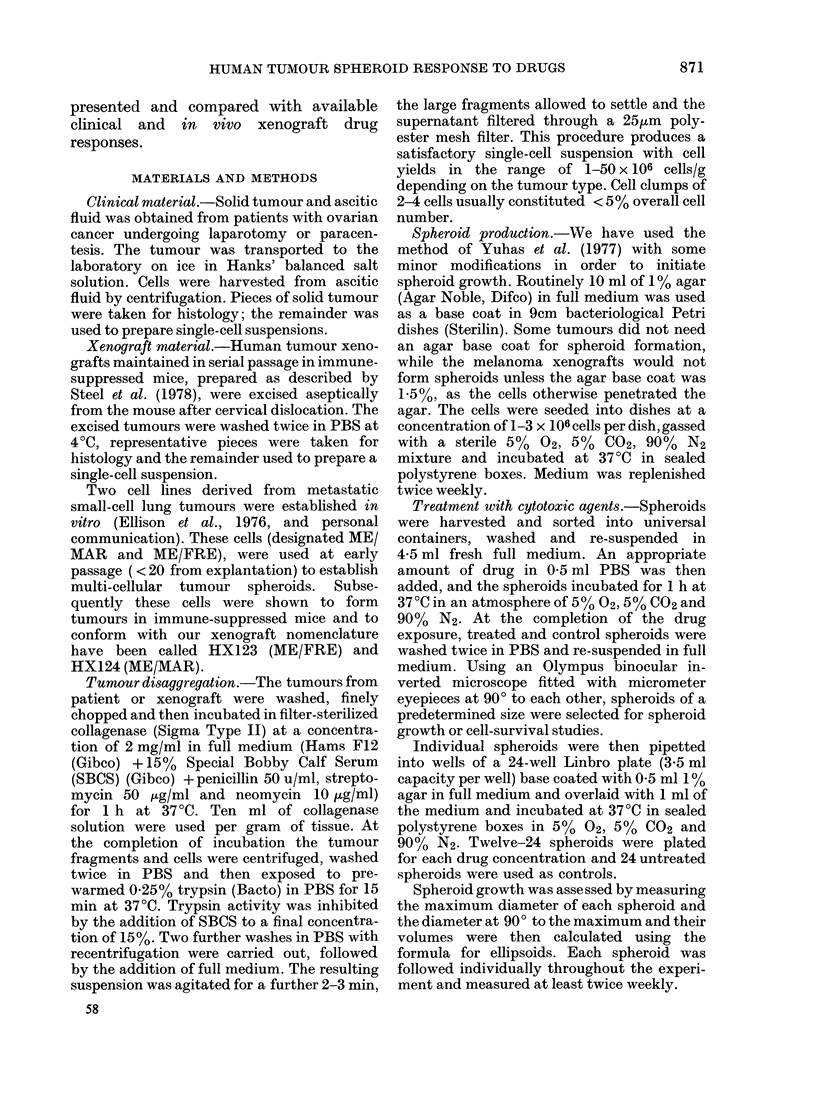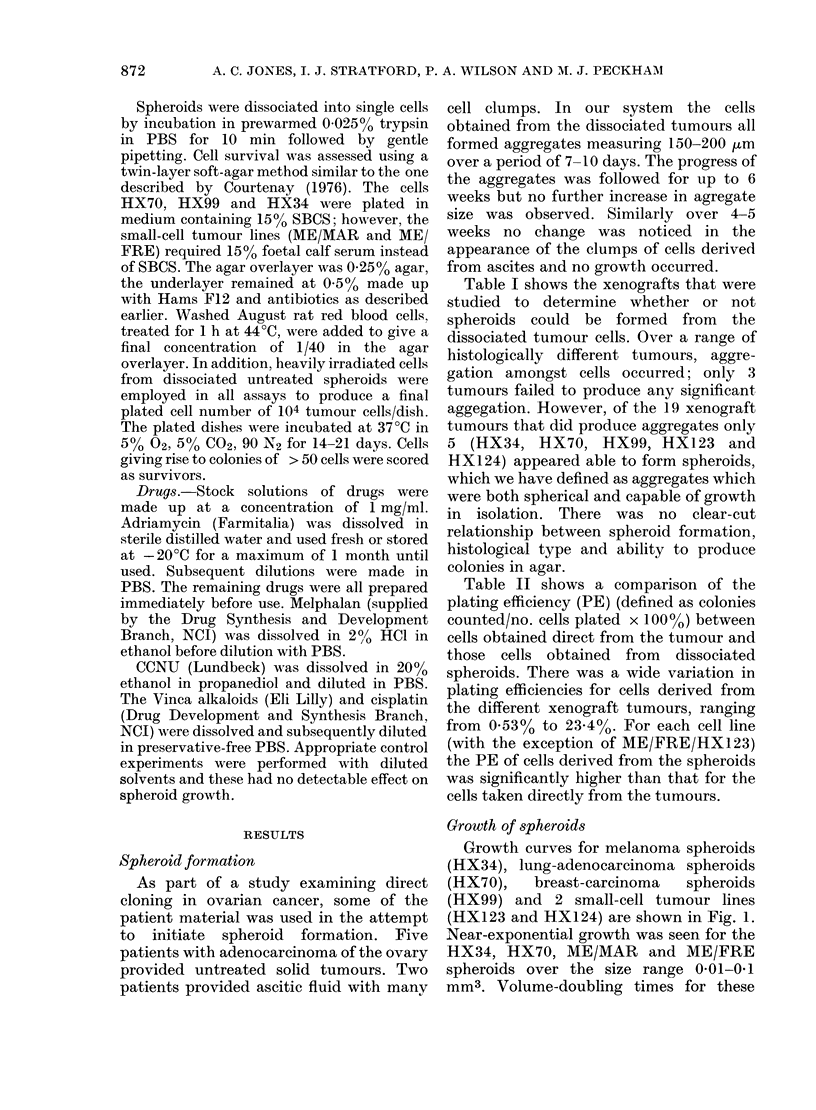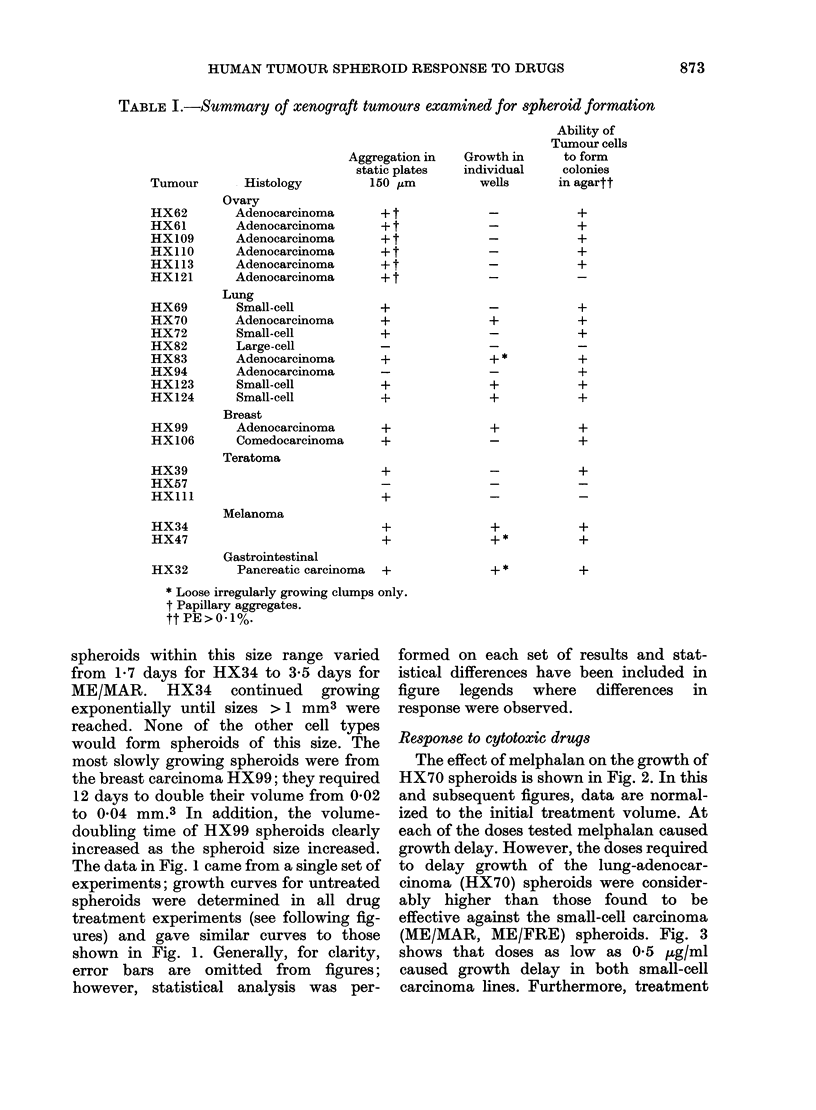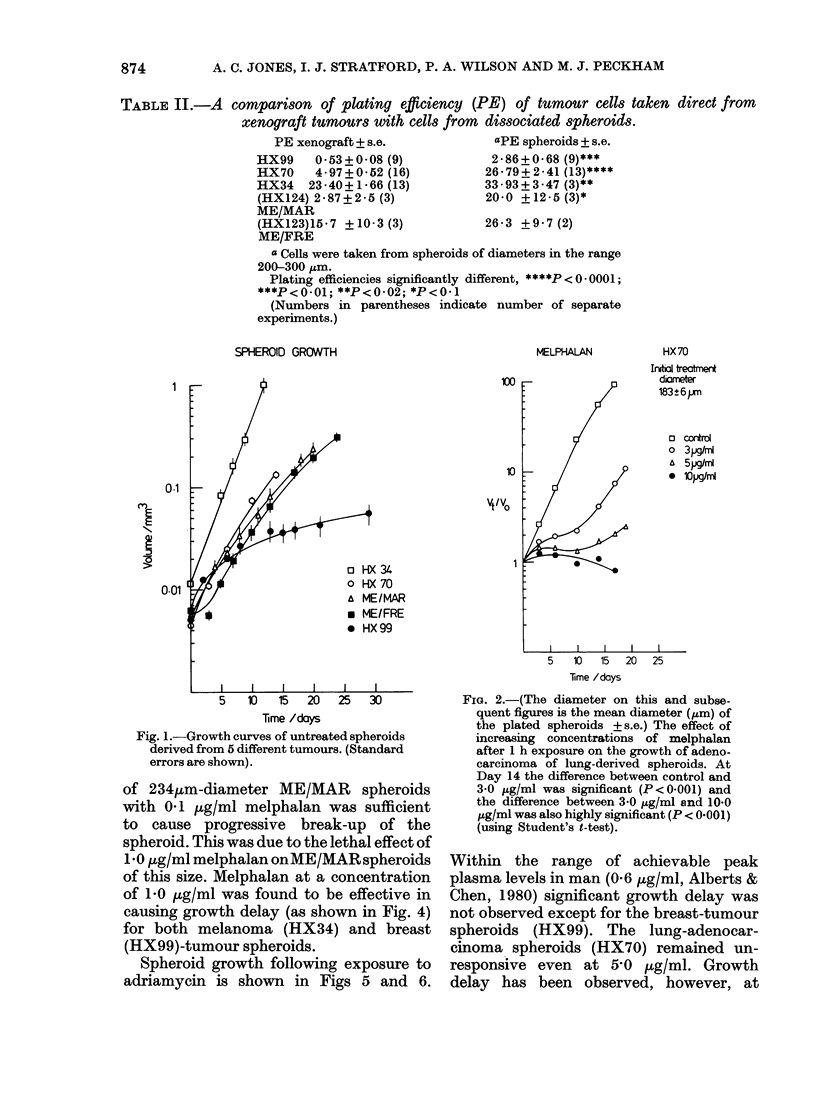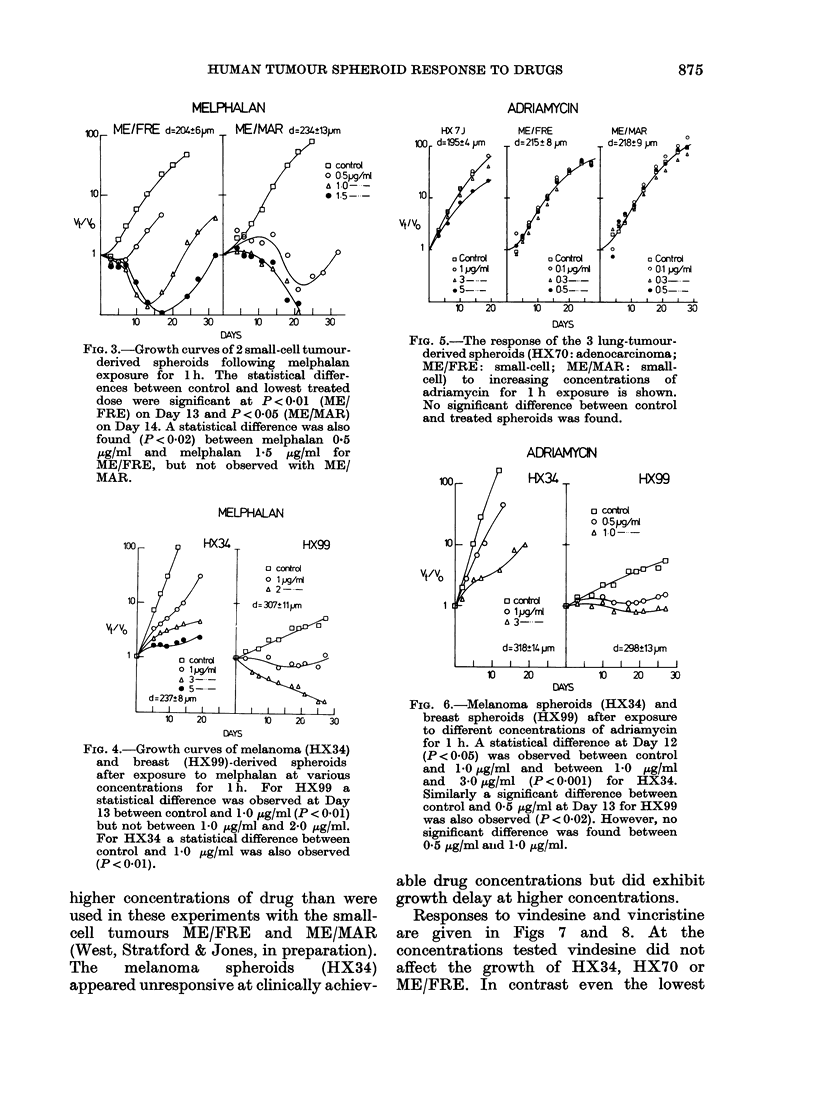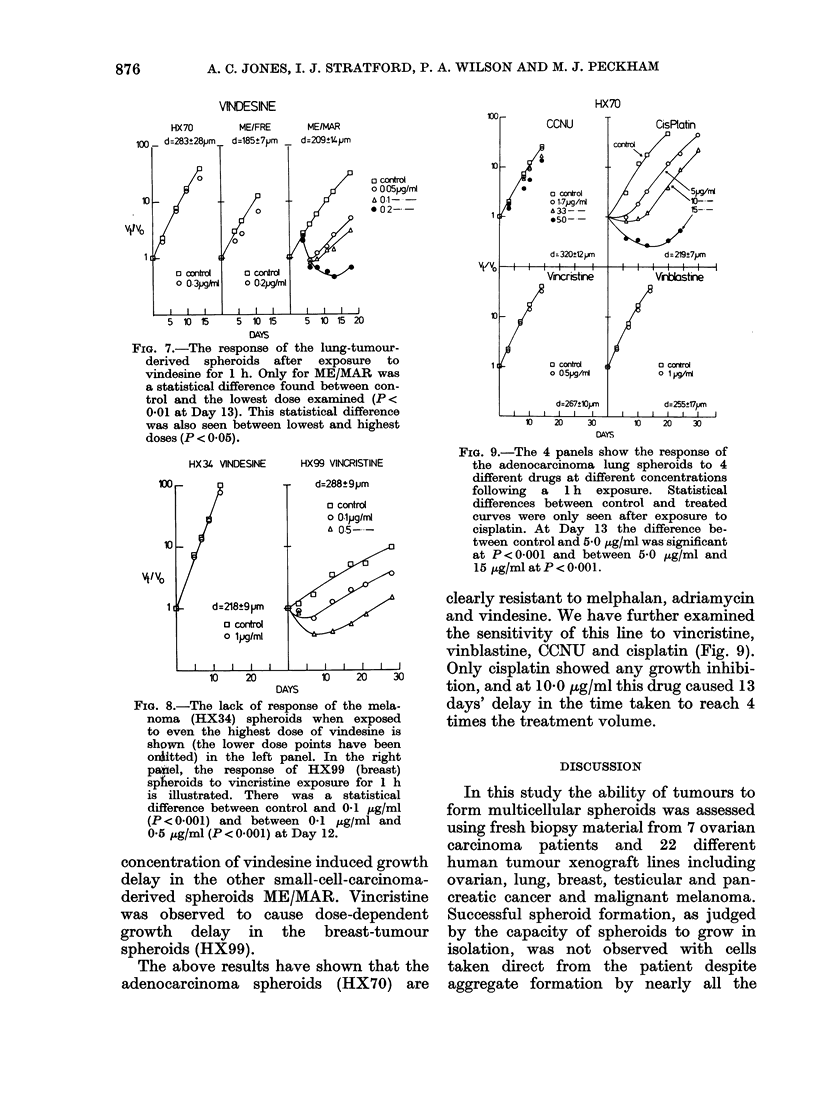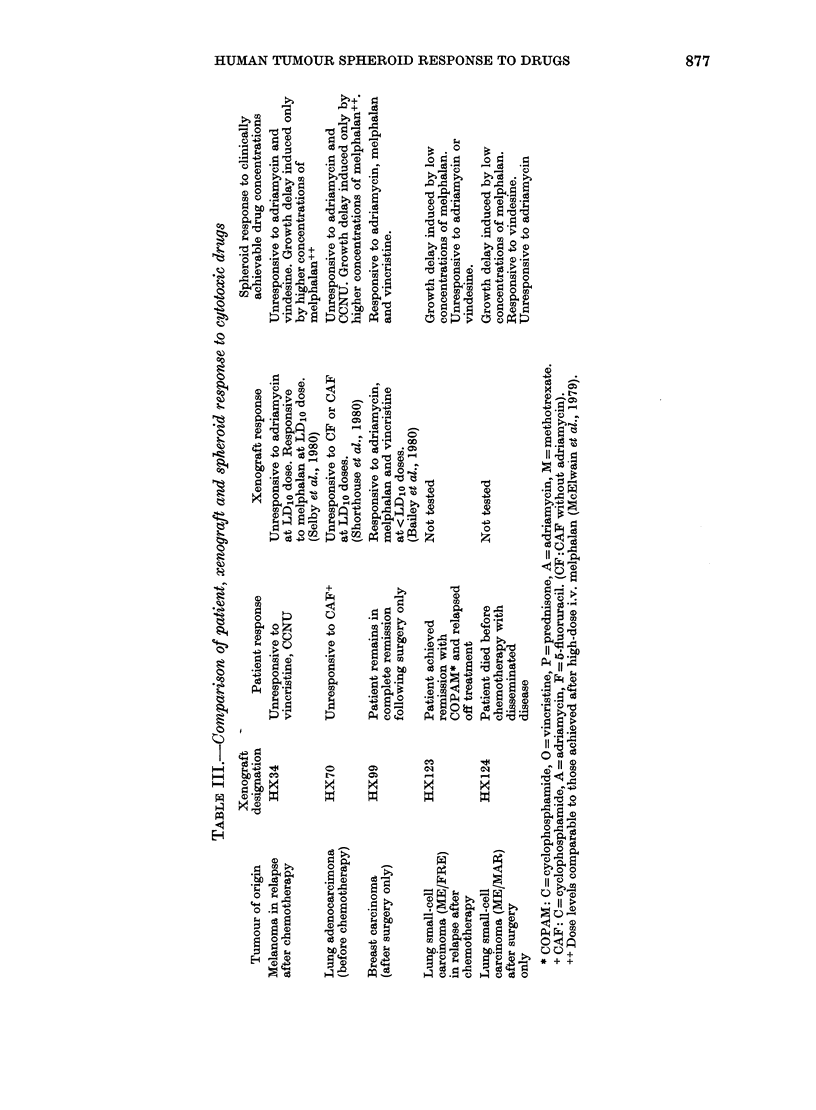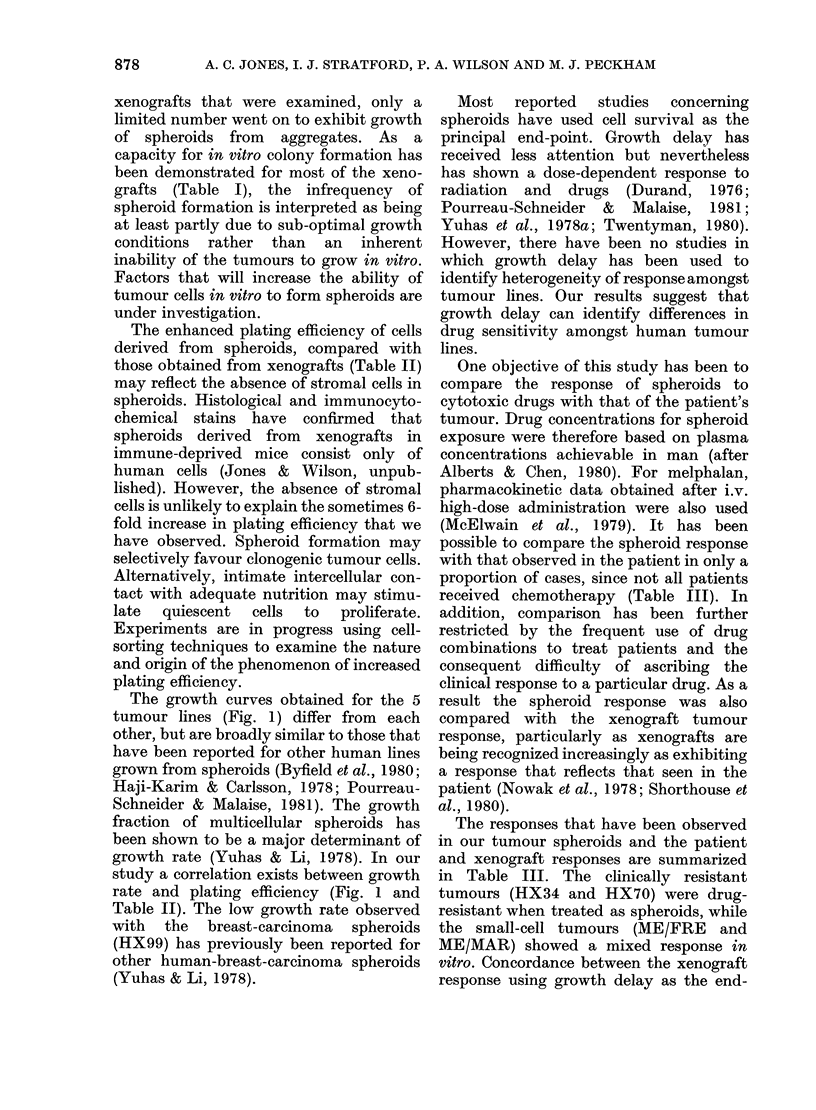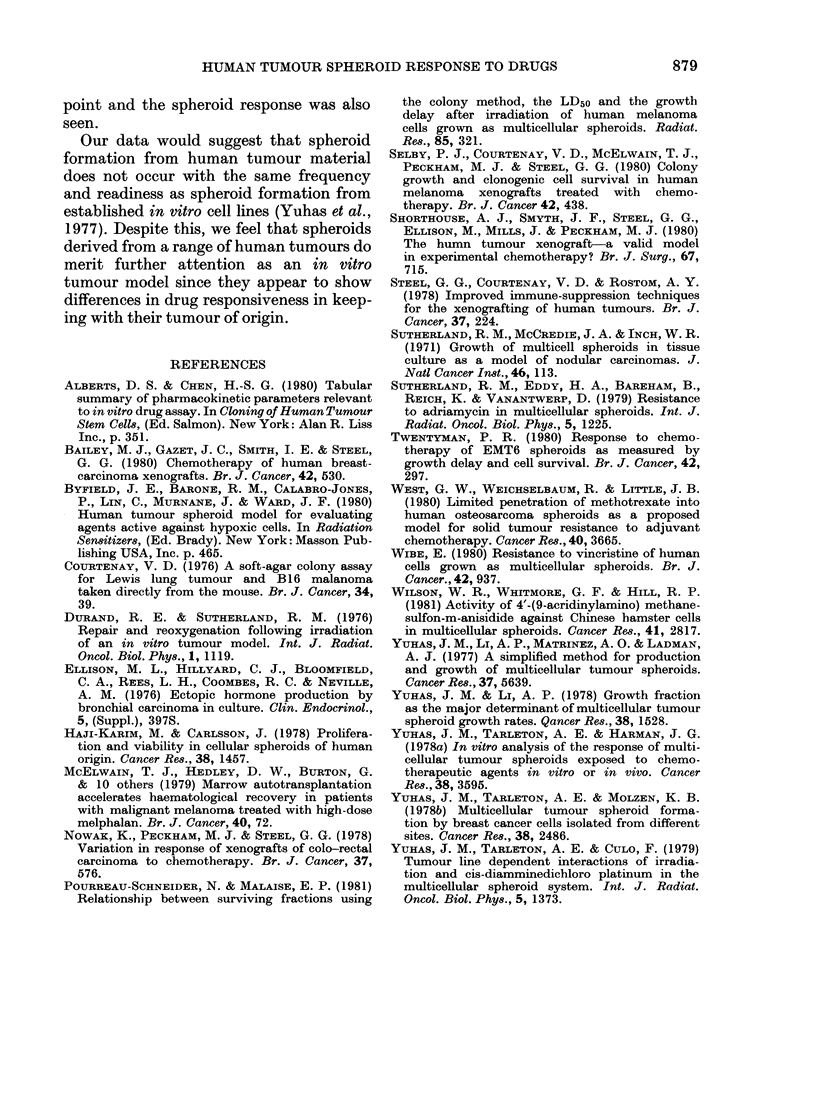# In vitro cytotoxic drug sensitivity testing of human tumour xenografts grown as multicellular tumour spheroids.

**DOI:** 10.1038/bjc.1982.296

**Published:** 1982-12

**Authors:** A. C. Jones, I. J. Stratford, P. A. Wilson, M. J. Peckham

## Abstract

Tumour cells from 7 patients with ovarian carcinoma and from 22 different human tumour xenografts representing a wide range of histological sub-types have been examined for multicellular spheroid forming ability. Spheroid formation was limited to cells derived from xenografts. Of the 22 lines tested, 5 formed spheroids capable of growth in isolation. There was no clear relationship between histological type and spheroid-forming ability. The plating efficiency of tumour cells obtained from spheroids was always greater than for the cells obtained from the dissociated tumour of origin and was in some cases as much as 6-fold greater. Spheroid growth was nearly exponential for 4 cell lines. Volume growth delay was used to investigate the activity of melphalan, adriamycin, the Vinca alkaloids, CCNU and cisplatin. Differences between lines in drug response broadly reflected patient and in vivo xenograft response.


*Br. J. Cancer* (1982) **46**, 870

### *IN VITRO* CYTOTOXIC DRUG SENSITIVITY TESTING OF HUMAN TUMOUR XENOGRAFTS GROWN AS MULTICELLULAR TUMOUR SPHEROIDS

A. C. JONES, I. J. STRATFORD, P. A. WILSON AND M. J. PECKHAM

*From the Radiotherapy Research Unit and Physics Department, Institute of Cancer Research, Clifton Avenue, Sutton, Surrey SM2 5PX*

Received 26 April 1982 Accepted 17 August 1982

**Summary.—Tumour cells from 7 patients with ovarian carcinoma and from 22 different human tumour xenografts representing a wide range of histological sub-types have been examined for multicellular spheroid forming ability. Spheroid formation was limited to cells derived from xenografts. Of the 22 lines tested, 5 formed spheroids capable of growth in isolation. There was no clear relationship between histological type and spheroid-forming ability. The plating efficiency of tumour cells obtained from spheroids was always greater than for the cells obtained from the dissociated tumour of origin and was in some cases as much as 6-fold greater. Spheroid growth was nearly exponential for 4 cell lines.**

**Volume growth delay was used to investigate the activity of melphalan, adriamycin, the Vinca alkaloids, CCNU and cisplatin. Differences between lines in drug response broadly reflected patient and *in vivo* xenograft response.**

THE CHARACTERISTICS of multicellular spheroids which include intimate cell-to-cell contact and diffusion gradients for oxygen, glucose and other nutrients, makes this a potentially interesting *in vitro* model for small solid tumours.

Chinese hamster V79 multicellular spheroids were first developed by Sutherland *et al.* (1971) and subsequently shown to have a number of properties characteristic of radiobiological behaviour *in vivo*, including the presence of hypoxic cells and the ability to re-oxygenate (Durand & Sutherland, 1976). More recently the spheroid has been used to investigate cellular responses to chemotherapy (Yuhas *et al.*, 1978*a*; Sutherland *et al.*, 1979; Twentyman, 1980; Wibe, 1980; West *et al.*, 1980). It is evident that some of the characteristics of spheroids, such as diffusion gradients (Sutherland *et al.*, 1979; West *et al.*, 1980; Wilson *et al.*, 1981) and the ability to recover from potentially lethal damage (PLD) after drug exposure (Twentyman, 1980) can profoundly influence chemotherapeutic response.

A simplified method of spheroid production using a static culture technique was described by Yuhas *et al.* (1977). Subsequently cells from a variety of sources, including some of human origin, were shown to form spheroids and to grow in culture (Yuhas *et al.*, 1978*b*; Haji-Karim & Carlsson, 1978; Pourreau-Schneider & Malaise, 1981). Differences in cell proliferation kinetics (Yuhas & Li, 1978) and chemosensitivity response (Yuhas *et al.*, 1979) between multicellular spheroids of different origin have been demonstrated. However, to date there has been no systematic published study which has examined the readiness with which cells from a range of different human tumour types will form spheroids. In this paper we report our studies of spheroid formation using cells derived from 6 different tumour types. Preliminary data on the response of spheroids to a range of cytotoxic drugs is presented and compared with available clinical and *in vivo* xenograft drug responses.

### MATERIALS AND METHODS

*Clinical material.*—Solid tumour and ascitic fluid was obtained from patients with ovarian cancer undergoing laparotomy or paracentesis. The tumour was transported to the laboratory on ice in Hanks' balanced salt solution. Cells were harvested from ascitic fluid by centrifugation. Pieces of solid tumour were taken for histology; the remainder was used to prepare single-cell suspensions.

*Xenograft material.*—Human tumour xenografts maintained in serial passage in immune-suppressed mice, prepared as described by Steel *et al.* (1978), were excised aseptically from the mouse after cervical dislocation. The excised tumours were washed twice in PBS at 4°C, representative pieces were taken for histology and the remainder used to prepare a single-cell suspension.

Two cell lines derived from metastatic small-cell lung tumours were established *in vitro* (Ellison *et al.*, 1976, and personal communication). These cells (designated ME/MAR and ME/FRE), were used at early passage (< 20 from explantation) to establish multi-cellular tumour spheroids. Subsequently these cells were shown to form tumours in immune-suppressed mice and to conform with our xenograft nomenclature have been called HX123 (ME/FRE) and HX124 (ME/MAR).

*Tumour disaggregation.*—The tumours from patient or xenograft were washed, finely chopped and then incubated in filter-sterilized collagenase (Sigma Type II) at a concentration of 2 mg/ml in full medium (Hams F12 (Gibco) +15% Special Bobby Calf Serum (SBCS) (Gibco) +penicillin 50 u/ml, streptomycin 50 μg/ml and neomycin 10 μg/ml) for 1 h at 37°C. Ten ml of collagenase solution were used per gram of tissue. At the completion of incubation the tumour fragments and cells were centrifuged, washed twice in PBS and then exposed to pre-warmed 0·25% trypsin (Bacto) in PBS for 15 min at 37°C. Trypsin activity was inhibited by the addition of SBCS to a final concentration of 15%. Two further washes in PBS with recentrifugation were carried out, followed by the addition of full medium. The resulting suspension was agitated for a further 2–3 min, the large fragments allowed to settle and the supernatant filtered through a 25μm polyester mesh filter. This procedure produces a satisfactory single-cell suspension with cell yields in the range of $1\text{–}50 \times 10^6$ cells/g depending on the tumour type. Cell clumps of 2–4 cells usually constituted < 5% overall cell number.

*Spheroid production.*—We have used the method of Yuhas *et al.* (1977) with some minor modifications in order to initiate spheroid growth. Routinely 10 ml of 1% agar (Agar Noble, Difco) in full medium was used as a base coat in 9cm bacteriological Petri dishes (Sterilin). Some tumours did not need an agar base coat for spheroid formation, while the melanoma xenografts would not form spheroids unless the agar base coat was 1·5%, as the cells otherwise penetrated the agar. The cells were seeded into dishes at a concentration of $1\text{–}3 \times 10^6$ cells per dish, gassed with a sterile 5% $O_2$, 5% $CO_2$, 90% $N_2$ mixture and incubated at 37°C in sealed polystyrene boxes. Medium was replenished twice weekly.

*Treatment with cytotoxic agents.*—Spheroids were harvested and sorted into universal containers, washed and re-suspended in 4·5 ml fresh full medium. An appropriate amount of drug in 0·5 ml PBS was then added, and the spheroids incubated for 1 h at 37°C in an atmosphere of 5% $O_2$, 5% $CO_2$ and 90% $N_2$. At the completion of the drug exposure, treated and control spheroids were washed twice in PBS and re-suspended in full medium. Using an Olympus binocular inverted microscope fitted with micrometer eyepieces at 90° to each other, spheroids of a predetermined size were selected for spheroid growth or cell-survival studies.

Individual spheroids were then pipetted into wells of a 24-well Linbro plate (3·5 ml capacity per well) base coated with 0·5 ml 1% agar in full medium and overlaid with 1 ml of the medium and incubated at 37°C in sealed polystyrene boxes in 5% $O_2$, 5% $CO_2$ and 90% $N_2$. Twelve–24 spheroids were plated for each drug concentration and 24 untreated spheroids were used as controls.

Spheroid growth was assessed by measuring the maximum diameter of each spheroid and the diameter at 90° to the maximum and their volumes were then calculated using the formula for ellipsoids. Each spheroid was followed individually throughout the experiment and measured at least twice weekly.

Spheroids were dissociated into single cells by incubation in prewarmed 0·025% trypsin in PBS for 10 min followed by gentle pipetting. Cell survival was assessed using a twin-layer soft-agar method similar to the one described by Courtenay (1976). The cells HX70, HX99 and HX34 were plated in medium containing 15% SBCS; however, the small-cell tumour lines (ME/MAR and ME/FRE) required 15% foetal calf serum instead of SBCS. The agar overlayer was 0·25% agar, the underlayer remained at 0·5% made up with Hams F12 and antibiotics as described earlier. Washed August rat red blood cells, treated for 1 h at 44°C, were added to give a final concentration of 1/40 in the agar overlayer. In addition, heavily irradiated cells from dissociated untreated spheroids were employed in all assays to produce a final plated cell number of $10^4$ tumour cells/dish. The plated dishes were incubated at 37°C in 5% $O_2$, 5% $CO_2$, 90 $N_2$ for 14–21 days. Cells giving rise to colonies of $>50$ cells were scored as survivors.

*Drugs.*—Stock solutions of drugs were made up at a concentration of 1 mg/ml. Adriamycin (Farmitalia) was dissolved in sterile distilled water and used fresh or stored at −20°C for a maximum of 1 month until used. Subsequent dilutions were made in PBS. The remaining drugs were all prepared immediately before use. Melphalan (supplied by the Drug Synthesis and Development Branch, NCI) was dissolved in 2% HCl in ethanol before dilution with PBS.

CCNU (Lundbeck) was dissolved in 20% ethanol in propanediol and diluted in PBS. The Vinca alkaloids (Eli Lilly) and cisplatin (Drug Development and Synthesis Branch, NCI) were dissolved and subsequently diluted in preservative-free PBS. Appropriate control experiments were performed with diluted solvents and these had no detectable effect on spheroid growth.

### RESULTS

### *Spheroid formation*

As part of a study examining direct cloning in ovarian cancer, some of the patient material was used in the attempt to initiate spheroid formation. Five patients with adenocarcinoma of the ovary provided untreated solid tumours. Two patients provided ascitic fluid with many cell clumps. In our system the cells obtained from the dissociated tumours all formed aggregates measuring 150–200 μm over a period of 7–10 days. The progress of the aggregates was followed for up to 6 weeks but no further increase in agregate size was observed. Similarly over 4–5 weeks no change was noticed in the appearance of the clumps of cells derived from ascites and no growth occurred.

Table I shows the xenografts that were studied to determine whether or not spheroids could be formed from the dissociated tumour cells. Over a range of histologically different tumours, aggregation amongst cells occurred; only 3 tumours failed to produce any significant aggegation. However, of the 19 xenograft tumours that did produce aggregates only 5 (HX34, HX70, HX99, HX123 and HX124) appeared able to form spheroids, which we have defined as aggregates which were both spherical and capable of growth in isolation. There was no clear-cut relationship between spheroid formation, histological type and ability to produce colonies in agar.

Table II shows a comparison of the plating efficiency (PE) (defined as colonies counted/no. cells plated ×100%) between cells obtained direct from the tumour and those cells obtained from dissociated spheroids. There was a wide variation in plating efficiencies for cells derived from the different xenograft tumours, ranging from 0·53% to 23·4%. For each cell line (with the exception of ME/FRE/HX123) the PE of cells derived from the spheroids was significantly higher than that for the cells taken directly from the tumours.

### *Growth of spheroids*

Growth curves for melanoma spheroids (HX34), lung-adenocarcinoma spheroids (HX70), breast-carcinoma spheroids (HX99) and 2 small-cell tumour lines (HX123 and HX124) are shown in Fig. 1. Near-exponential growth was seen for the HX34, HX70, ME/MAR and ME/FRE spheroids over the size range 0·01–0·1 $mm^3$. Volume-doubling times for these

TABLE I.—*Summary of xenograft tumours examined for spheroid formation*

| Tumour | Histology | Aggregation in static plates 150 μm | Growth in individual wells | Ability of Tumour cells to form colonies in agar†† |
|---|---|---|---|---|
| | Ovary | | | |
| HX62 | Adenocarcinoma | +† | − | + |
| HX61 | Adenocarcinoma | +† | − | + |
| HX109 | Adenocarcinoma | +† | − | + |
| HX110 | Adenocarcinoma | +† | − | + |
| HX113 | Adenocarcinoma | +† | − | + |
| HX121 | Adenocarcinoma | +† | − | − |
| | Lung | | | |
| HX69 | Small-cell | + | − | + |
| HX70 | Adenocarcinoma | + | + | + |
| HX72 | Small-cell | + | − | + |
| HX82 | Large-cell | − | − | − |
| HX83 | Adenocarcinoma | + | +* | + |
| HX94 | Adenocarcinoma | − | − | + |
| HX123 | Small-cell | + | + | + |
| HX124 | Small-cell | + | + | + |
| | Breast | | | |
| HX99 | Adenocarcinoma | + | + | + |
| HX106 | Comedocarcinoma | + | − | + |
| | Teratoma | | | |
| HX39 | | + | − | + |
| HX57 | | − | − | − |
| HX111 | | + | − | − |
| | Melanoma | | | |
| HX34 | | + | + | + |
| HX47 | | + | +* | + |
| | Gastrointestinal | | | |
| HX32 | Pancreatic carcinoma | + | +* | + |

* Loose irregularly growing clumps only.

† Papillary aggregates.

†† PE > 0·1%.

spheroids within this size range varied from 1·7 days for HX34 to 3·5 days for ME/MAR. HX34 continued growing exponentially until sizes > 1 $mm^3$ were reached. None of the other cell types would form spheroids of this size. The most slowly growing spheroids were from the breast carcinoma HX99; they required 12 days to double their volume from 0·02 to 0·04 $mm.^3$ In addition, the volume-doubling time of HX99 spheroids clearly increased as the spheroid size increased. The data in Fig. 1 came from a single set of experiments; growth curves for untreated spheroids were determined in all drug treatment experiments (see following figures) and gave similar curves to those shown in Fig. 1. Generally, for clarity, error bars are omitted from figures; however, statistical analysis was performed on each set of results and statistical differences have been included in figure legends where differences in response were observed.

*Response to cytotoxic drugs*

The effect of melphalan on the growth of HX70 spheroids is shown in Fig. 2. In this and subsequent figures, data are normalized to the initial treatment volume. At each of the doses tested melphalan caused growth delay. However, the doses required to delay growth of the lung-adenocarcinoma (HX70) spheroids were considerably higher than those found to be effective against the small-cell carcinoma (ME/MAR, ME/FRE) spheroids. Fig. 3 shows that doses as low as 0·5 μg/ml caused growth delay in both small-cell carcinoma lines. Furthermore, treatment

TABLE II.—*A comparison of plating efficiency (PE) of tumour cells taken direct from xenograft tumours with cells from dissociated spheroids.*

| PE xenograft ± s.e. | | [a]PE spheroids ± s.e. |
|---|---|---|
| HX99 | 0·53 ± 0·08 (9) | 2·86 ± 0·68 (9)*** |
| HX70 | 4·97 ± 0·52 (16) | 26·79 ± 2·41 (13)**** |
| HX34 | 23·40 ± 1·66 (13) | 33·93 ± 3·47 (3)** |
| (HX124) ME/MAR | 2·87 ± 2·5 (3) | 20·0 ± 12·5 (3)* |
| (HX123) ME/FRE | 15·7 ± 10·3 (3) | 26·3 ± 9·7 (2) |

[a] Cells were taken from spheroids of diameters in the range 200–300 μm.

Plating efficiencies significantly different, ****$P<0\cdot0001$; ***$P<0\cdot01$; **$P<0\cdot02$; *$P<0\cdot1$

(Numbers in parentheses indicate number of separate experiments.)

Fig. 1.—Growth curves of untreated spheroids derived from 5 different tumours. (Standard errors are shown).

FIG. 2.—(The diameter on this and subsequent figures is the mean diameter (μm) of the plated spheroids ± s.e.) The effect of increasing concentrations of melphalan after 1 h exposure on the growth of adenocarcinoma of lung-derived spheroids. At Day 14 the difference between control and 3·0 μg/ml was significant ($P<0\cdot001$) and the difference between 3·0 μg/ml and 10·0 μg/ml was also highly significant ($P<0\cdot001$) (using Student's *t*-test).

of 234μm-diameter ME/MAR spheroids with 0·1 μg/ml melphalan was sufficient to cause progressive break-up of the spheroid. This was due to the lethal effect of 1·0 μg/ml melphalan on ME/MAR spheroids of this size. Melphalan at a concentration of 1·0 μg/ml was found to be effective in causing growth delay (as shown in Fig. 4) for both melanoma (HX34) and breast (HX99)-tumour spheroids.

Spheroid growth following exposure to adriamycin is shown in Figs 5 and 6. Within the range of achievable peak plasma levels in man (0·6 μg/ml, Alberts & Chen, 1980) significant growth delay was not observed except for the breast-tumour spheroids (HX99). The lung-adenocarcinoma spheroids (HX70) remained unresponsive even at 5·0 μg/ml. Growth delay has been observed, however, at

FIG. 3.—Growth curves of 2 small-cell tumour-derived spheroids following melphalan exposure for 1 h. The statistical differences between control and lowest treated dose were significant at $P<0.01$ (ME/FRE) on Day 13 and $P<0.05$ (ME/MAR) on Day 14. A statistical difference was also found ($P<0.02$) between melphalan 0·5 μg/ml and melphalan 1·5 μg/ml for ME/FRE, but not observed with ME/MAR.

FIG. 5.—The response of the 3 lung-tumour-derived spheroids (HX70: adenocarcinoma; ME/FRE: small-cell; ME/MAR: small-cell) to increasing concentrations of adriamycin for 1 h exposure is shown. No significant difference between control and treated spheroids was found.

FIG. 4.—Growth curves of melanoma (HX34) and breast (HX99)-derived spheroids after exposure to melphalan at various concentrations for 1 h. For HX99 a statistical difference was observed at Day 13 between control and 1·0 μg/ml ($P<0.01$) but not between 1·0 μg/ml and 2·0 μg/ml. For HX34 a statistical difference between control and 1·0 μg/ml was also observed ($P<0.01$).

FIG. 6.—Melanoma spheroids (HX34) and breast spheroids (HX99) after exposure to different concentrations of adriamycin for 1 h. A statistical difference at Day 12 ($P<0.05$) was observed between control and 1·0 μg/ml and between 1·0 μg/ml and 3·0 μg/ml ($P<0.001$) for HX34. Similarly a significant difference between control and 0·5 μg/ml at Day 13 for HX99 was also observed ($P<0.02$). However, no significant difference was found between 0·5 μg/ml and 1·0 μg/ml.

higher concentrations of drug than were used in these experiments with the small-cell tumours ME/FRE and ME/MAR (West, Stratford & Jones, in preparation). The melanoma spheroids (HX34) appeared unresponsive at clinically achievable drug concentrations but did exhibit growth delay at higher concentrations.

Responses to vindesine and vincristine are given in Figs 7 and 8. At the concentrations tested vindesine did not affect the growth of HX34, HX70 or ME/FRE. In contrast even the lowest

FIG. 7.—The response of the lung-tumour-derived spheroids after exposure to vindesine for 1 h. Only for ME/MAR was a statistical difference found between control and the lowest dose examined ($P < 0.01$ at Day 13). This statistical difference was also seen between lowest and highest doses ($P < 0.05$).

FIG. 8.—The lack of response of the melanoma (HX34) spheroids when exposed to even the highest dose of vindesine is shown (the lower dose points have been omitted) in the left panel. In the right panel, the response of HX99 (breast) spheroids to vincristine exposure for 1 h is illustrated. There was a statistical difference between control and 0·1 μg/ml ($P < 0.001$) and between 0·1 μg/ml and 0·5 μg/ml ($P < 0.001$) at Day 12.

FIG. 9.—The 4 panels show the response of the adenocarcinoma lung spheroids to 4 different drugs at different concentrations following a 1 h exposure. Statistical differences between control and treated curves were only seen after exposure to cisplatin. At Day 13 the difference between control and 5·0 μg/ml was significant at $P < 0.001$ and between 5·0 μg/ml and 15 μg/ml at $P < 0.001$.

concentration of vindesine induced growth delay in the other small-cell-carcinoma-derived spheroids ME/MAR. Vincristine was observed to cause dose-dependent growth delay in the breast-tumour spheroids (HX99).

The above results have shown that the adenocarcinoma spheroids (HX70) are clearly resistant to melphalan, adriamycin and vindesine. We have further examined the sensitivity of this line to vincristine, vinblastine, CCNU and cisplatin (Fig. 9). Only cisplatin showed any growth inhibition, and at 10·0 μg/ml this drug caused 13 days' delay in the time taken to reach 4 times the treatment volume.

### DISCUSSION

In this study the ability of tumours to form multicellular spheroids was assessed using fresh biopsy material from 7 ovarian carcinoma patients and 22 different human tumour xenograft lines including ovarian, lung, breast, testicular and pancreatic cancer and malignant melanoma. Successful spheroid formation, as judged by the capacity of spheroids to grow in isolation, was not observed with cells taken direct from the patient despite aggregate formation by nearly all the

TABLE III.—*Comparison of patient, xenograft and spheroid response to cytotoxic drugs*

| Tumour of origin | Xenograft designation | Patient response | Xenograft response | Spheroid response to clinically achievable drug concentrations |
|---|---|---|---|---|
| Melanoma in relapse after chemotherapy | HX34 | Unresponsive to vincristine, CCNU | Unresponsive to adriamycin at $LD_{10}$ dose. Responsive to melphalan at $LD_{10}$ dose. (Selby *et al.*, 1980) | Unresponsive to adriamycin and vindesine. Growth delay induced only by higher concentrations of melphalan++ |
| Lung adenocarcimona (before chemotherapy) | HX70 | Unresponsive to CAF+ | Unresponsive to CF or CAF at $LD_{10}$ doses. (Shorthouse *et al.*, 1980) | Unresponsive to adriamycin and CCNU. Growth delay induced only by higher concentrations of melphalan++. |
| Breast carcinoma (after surgery only) | HX99 | Patient remains in complete remission following surgery only | Responsive to adriamycin, melphalan and vincristine at $< LD_{10}$ doses. (Bailey *et al.*, 1980) | Responsive to adriamycin, melphalan and vincristine. |
| Lung small-cell carcinoma (ME/FRE) in relapse after chemotherapy | HX123 | Patient achieved remission with COPAM* and relapsed off treatment | Not tested | Growth delay induced by low concentrations of melphalan. Unresponsive to adriamycin or vindesine. |
| Lung small-cell carcinoma (ME/MAR) after surgery only | HX124 | Patient died before chemotherapy with disseminated disease | Not tested | Growth delay induced by low concentrations of melphalan. Responsive to vindesine. Unresponsive to adriamycin |

* COPAM: C = cyclophosphamide, O = vincristine, P = prednisone, A = adriamycin, M = methotrexate.

+ CAF: C = cyclophosphamide, A = adriamycin, F = 5-fluoruracil. (CF:CAF without adriamycin).

++ Dose levels comparable to those achieved after high-dose i.v. melphalan (McElwain *et al.*, 1979).

xenografts that were examined, only a limited number went on to exhibit growth of spheroids from aggregates. As a capacity for *in vitro* colony formation has been demonstrated for most of the xenografts (Table I), the infrequency of spheroid formation is interpreted as being at least partly due to sub-optimal growth conditions rather than an inherent inability of the tumours to grow *in vitro*. Factors that will increase the ability of tumour cells *in vitro* to form spheroids are under investigation.

The enhanced plating efficiency of cells derived from spheroids, compared with those obtained from xenografts (Table II) may reflect the absence of stromal cells in spheroids. Histological and immunocytochemical stains have confirmed that spheroids derived from xenografts in immune-deprived mice consist only of human cells (Jones & Wilson, unpublished). However, the absence of stromal cells is unlikely to explain the sometimes 6-fold increase in plating efficiency that we have observed. Spheroid formation may selectively favour clonogenic tumour cells. Alternatively, intimate intercellular contact with adequate nutrition may stimulate quiescent cells to proliferate. Experiments are in progress using cell-sorting techniques to examine the nature and origin of the phenomenon of increased plating efficiency.

The growth curves obtained for the 5 tumour lines (Fig. 1) differ from each other, but are broadly similar to those that have been reported for other human lines grown from spheroids (Byfield *et al.*, 1980; Haji-Karim & Carlsson, 1978; Pourreau-Schneider & Malaise, 1981). The growth fraction of multicellular spheroids has been shown to be a major determinant of growth rate (Yuhas & Li, 1978). In our study a correlation exists between growth rate and plating efficiency (Fig. 1 and Table II). The low growth rate observed with the breast-carcinoma spheroids (HX99) has previously been reported for other human-breast-carcinoma spheroids (Yuhas & Li, 1978).

Most reported studies concerning spheroids have used cell survival as the principal end-point. Growth delay has received less attention but nevertheless has shown a dose-dependent response to radiation and drugs (Durand, 1976; Pourreau-Schneider & Malaise, 1981; Yuhas *et al.*, 1978*a*; Twentyman, 1980). However, there have been no studies in which growth delay has been used to identify heterogeneity of response amongst tumour lines. Our results suggest that growth delay can identify differences in drug sensitivity amongst human tumour lines.

One objective of this study has been to compare the response of spheroids to cytotoxic drugs with that of the patient's tumour. Drug concentrations for spheroid exposure were therefore based on plasma concentrations achievable in man (after Alberts & Chen, 1980). For melphalan, pharmacokinetic data obtained after i.v. high-dose administration were also used (McElwain *et al.*, 1979). It has been possible to compare the spheroid response with that observed in the patient in only a proportion of cases, since not all patients received chemotherapy (Table III). In addition, comparison has been further restricted by the frequent use of drug combinations to treat patients and the consequent difficulty of ascribing the clinical response to a particular drug. As a result the spheroid response was also compared with the xenograft tumour response, particularly as xenografts are being recognized increasingly as exhibiting a response that reflects that seen in the patient (Nowak *et al.*, 1978; Shorthouse *et al.*, 1980).

The responses that have been observed in our tumour spheroids and the patient and xenograft responses are summarized in Table III. The clinically resistant tumours (HX34 and HX70) were drug-resistant when treated as spheroids, while the small-cell tumours (ME/FRE and ME/MAR) showed a mixed response *in vitro*. Concordance between the xenograft response using growth delay as the endpoint and the spheroid response was also seen.

Our data would suggest that spheroid formation from human tumour material does not occur with the same frequency and readiness as spheroid formation from established *in vitro* cell lines (Yuhas *et al.*, 1977). Despite this, we feel that spheroids derived from a range of human tumours do merit further attention as an *in vitro* tumour model since they appear to show differences in drug responsiveness in keeping with their tumour of origin.

### REFERENCES

ALBERTS, D. S. & CHEN, H.-S. G. (1980) Tabular summary of pharmacokinetic parameters relevant to *in vitro* drug assay. In *Cloning of Human Tumour Stem Cells*, (Ed. Salmon). New York: Alan R. Liss Inc., p. 351.

BAILEY, M. J., GAZET, J. C., SMITH, I. E. & STEEL, G. G. (1980) Chemotherapy of human breast-carcinoma xenografts. *Br. J. Cancer*, **42**, 530.

BYFIELD, J. E., BARONE, R. M., CALABRO-JONES, P., LIN, C., MURNANE, J. & WARD, J. F. (1980) Human tumour spheroid model for evaluating agents active against hypoxic cells. In *Radiation Sensitizers*, (Ed. Brady). New York: Masson Publishing USA, Inc. p. 465.

COURTENAY, V. D. (1976) A soft-agar colony assay for Lewis lung tumour and B16 malanoma taken directly from the mouse. *Br. J. Cancer*, **34**, 39.

DURAND, R. E. & SUTHERLAND, R. M. (1976) Repair and reoxygenation following irradiation of an *in vitro* tumour model. *Int. J. Radiat. Oncol. Biol. Phys.*, **1**, 1119.

ELLISON, M. L., HILLYARD, C. J., BLOOMFIELD, C. A., REES, L. H., COOMBES, R. C. & NEVILLE, A. M. (1976) Ectopic hormone production by bronchial carcinoma in culture. *Clin. Endocrinol.*, **5**, (Suppl.), 397S.

HAJI-KARIM, M. & CARLSSON, J. (1978) Proliferation and viability in cellular spheroids of human origin. *Cancer Res.*, **38**, 1457.

MCELWAIN, T. J., HEDLEY, D. W., BURTON, G. & 10 others (1979) Marrow autotransplantation accelerates haematological recovery in patients with malignant melanoma treated with high-dose melphalan. *Br. J. Cancer*, **40**, 72.

NOWAK, K., PECKHAM, M. J. & STEEL, G. G. (1978) Variation in response of xenografts of colo–rectal carcinoma to chemotherapy. *Br. J. Cancer*, **37**, 576.

POURREAU-SCHNEIDER, N. & MALAISE, E. P. (1981) Relationship between surviving fractions using the colony method, the $LD_{50}$ and the growth delay after irradiation of human melanoma cells grown as multicellular spheroids. *Radiat. Res.*, **85**, 321.

SELBY, P. J., COURTENAY, V. D., MCELWAIN, T. J., PECKHAM, M. J. & STEEL, G. G. (1980) Colony growth and clonogenic cell survival in human melanoma xenografts treated with chemotherapy. *Br. J. Cancer* **42**, 438.

SHORTHOUSE, A. J., SMYTH, J. F., STEEL, G. G., ELLISON, M., MILLS, J. & PECKHAM, M. J. (1980) The humn tumour xenograft—a valid model in experimental chemotherapy? *Br. J. Surg.*, **67**, 715.

STEEL, G. G., COURTENAY, V. D. & ROSTOM, A. Y. (1978) Improved immune-suppression techniques for the xenografting of human tumours. *Br. J. Cancer*, **37**, 224.

SUTHERLAND, R. M., MCCREDIE, J. A. & INCH, W. R. (1971) Growth of multicell spheroids in tissue culture as a model of nodular carcinomas. *J. Natl Cancer Inst.*, **46**, 113.

SUTHERLAND, R. M., EDDY, H. A., BAREHAM, B., REICH, K. & VANANTWERP, D. (1979) Resistance to adriamycin in multicellular spheroids. *Int. J. Radiat. Oncol. Biol. Phys.*, **5**, 1225.

TWENTYMAN, P. R. (1980) Response to chemotherapy of EMT6 spheroids as measured by growth delay and cell survival. *Br. J. Cancer*, **42**, 297.

WEST, G. W., WEICHSELBAUM, R. & LITTLE, J. B. (1980) Limited penetration of methotrexate into human osteosarcoma spheroids as a proposed model for solid tumour resistance to adjuvant chemotherapy. *Cancer Res.*, **40**, 3665.

WIBE, E. (1980) Resistance to vincristine of human cells grown as multicellular spheroids. *Br. J. Cancer.*, **42**, 937.

WILSON, W. R., WHITMORE, G. F. & HILL, R. P. (1981) Activity of 4′-(9-acridinylamino) methane-sulfon-m-anisidide against Chinese hamster cells in multicellular spheroids. *Cancer Res.*, **41**, 2817.

YUHAS, J. M., LI, A. P., MATRINEZ, A. O. & LADMAN, A. J. (1977) A simplified method for production and growth of multicellular tumour spheroids. *Cancer Res.*, **37**, 5639.

YUHAS, J. M. & LI, A. P. (1978) Growth fraction as the major determinant of multicellular tumour spheroid growth rates. *Qancer Res.*, **38**, 1528.

YUHAS, J. M., TARLETON, A. E. & HARMAN, J. G. (1978*a*) *In vitro* analysis of the response of multicellular tumour spheroids exposed to chemotherapeutic agents *in vitro* or *in vivo*. *Cancer Res.*, **38**, 3595.

YUHAS, J. M., TARLETON, A. E. & MOLZEN, K. B. (1978*b*) Multicellular tumour spheroid formation by breast cancer cells isolated from different sites. *Cancer Res.*, **38**, 2486.

YUHAS, J. M., TARLETON, A. E. & CULO, F. (1979) Tumour line dependent interactions of irradiation and cis-diamminedichloro platinum in the multicellular spheroid system. *Int. J. Radiat. Oncol. Biol. Phys.*, **5**, 1373.